# pH profiles of 3-chymotrypsin-like protease (3CLpro) from SARS-CoV-2 elucidate its catalytic mechanism and a histidine residue critical for activity

**DOI:** 10.1016/j.jbc.2022.102790

**Published:** 2022-12-09

**Authors:** Kenana Al Adem, Juliana C. Ferreira, Samar Fadl, Wael M. Rabeh

**Affiliations:** Science Division, New York University Abu Dhabi, Abu Dhabi, United Arab Emirates

**Keywords:** COVID-19, SARS-CoV-2, 3-chymotrypsin-like protease, Catalytic dyad, Conserved histidine, Thermodynamic stability, Initial velocity studies, pH studies, 3CLpro, 3-chymotrypsin-like protease, DMSO, dimethyl sulfoxide, DSC, differential scanning calorimetry, MD, molecular dynamics, β-ME, β-mercaptoethanol, nsp, nonstructural protein, PLpro, papain-like protease, SARS-CoV, severe acute respiratory syndrome coronavirus, SARS-CoV-2, severe acute respiratory syndrome coronavirus 2, TCEP, Tris(2-carboxyethyl)phosphine

## Abstract

3-Chymotrypsin-like protease (3CLpro) is a promising drug target for coronavirus disease 2019 and related coronavirus diseases because of the essential role of this protease in processing viral polyproteins after infection. Understanding the detailed catalytic mechanism of 3CLpro is essential for designing effective inhibitors of infection by severe acute respiratory syndrome coronavirus 2 (SARS-CoV-2). Molecular dynamics studies have suggested pH-dependent conformational changes of 3CLpro, but experimental pH profiles of SARS-CoV-2 3CLpro and analyses of the conserved active-site histidine residues have not been reported. In this work, pH-dependence studies of the kinetic parameters of SARS-CoV-2 3CLpro revealed a bell-shaped pH profile with 2 p*K*_a_ values (6.9 ± 0.1 and 9.4 ± 0.1) attributable to ionization of the catalytic dyad His41 and Cys145, respectively. Our investigation of the roles of conserved active-site histidines showed that different amino acid substitutions of His163 produced inactive enzymes, indicating a key role of His163 in maintaining catalytically active SARS-CoV-2 3CLpro. By contrast, the H164A and H172A mutants retained 75% and 26% of the activity of WT, respectively. The alternative amino acid substitutions H172K and H172R did not recover the enzymatic activity, whereas H172Y restored activity to a level similar to that of the WT enzyme. The pH profiles of H164A, H172A, and H172Y were similar to those of the WT enzyme, with comparable p*K*_a_ values for the catalytic dyad. Taken together, the experimental data support a general base mechanism of SARS-CoV-2 3CLpro and indicate that the neutral states of the catalytic dyad and active-site histidine residues are required for maximum enzyme activity.

Since the advent of the 21st century, the globe has experienced three epidemics caused by coronaviruses, the most recent of which is severe acute respiratory syndrome coronavirus 2 (SARS-CoV-2). SARS-CoV-2 first emerged among the Chinese population in the city of Wuhan in December 2019 before spreading worldwide at an exceptionally high rate. SARS-CoV-2 is responsible for coronavirus disease 2019 (COVID-19), which is characterized by influenza-like symptoms, such as fever, fatigue, diarrhea, dry cough, and shortness of breath. According to the World Health Organization, the global COVID-19 pandemic has resulted in over 600 million cases and six million reported deaths thus far (1, 2). COVID-19 is considered one of the most challenging viral outbreaks in contemporary times. Fortunately, the development of effective vaccines against SARS-CoV-2 has contributed to reducing viral transmission and preserving public health (3). However, as we continue into the third year of COVID-19 pandemic, additional effective antiviral treatments are urgently needed to combat current and newly emerging SARS-CoV-2 variants as well as future coronavirus outbreaks.

A coronavirus is a small spherical assembly with club-shaped protrusions composed of structural spike proteins that enable host cell entry (4). Once inside host cells, the coronavirus releases a single-stranded positive-sense RNA genome with 14 ORFs that encode 27 structural and nonstructural proteins (nsps) (2, 5). The 2 largest ORFs (ORF1a/b) are immediately translated by the host cell machinery into 2 overlapping polyproteins, pp1a and pp1ab, which encode nsps that are essential for the viral replication/transcription cycles (6, 7). These polyproteins are then cleaved by the highly conserved viral cysteine proteases, 3-chymotrypsin-like protease (3CLpro) and papain-like protease (PLpro). First, 3CLpro (nsp5) catalyzes its own cleavage at its N and C termini before liberating the other 11 nsps (nsp4–11/16) from the polyproteins. The remaining nsps (nsp1–4) are cleaved by PLpro (nsp3), which first autoprocesses its own cleavage (5, 8, 9, 10). The 2 cysteine proteases, 3CLpro and PLpro, are highly conserved among coronaviruses, including severe acute respiratory syndrome coronavirus (SARS-CoV) and Middle East respiratory syndrome coronavirus, which emerged in 2002 and 2012, respectively. Given that 3CLpro catalyzes the release of the majority of the nsps, this enzyme represents an attractive drug target for the development of effective and safe antivirals against COVID-19 and other coronavirus diseases (2, 11).

Previous studies have shown that homodimer formation is required for 3CLpro catalytic activity (12, 13, 14, 15, 16, 17, 18). However, we recently showed that dimerization does not necessarily guarantee a functional 3CLpro enzyme, as some mutations lead to complete inactivation of the enzyme without disrupting the dimer conformation (19). The crystal structure of 3CLpro revealed that the monomeric subunit comprises 3 domains, where domain I (residues 10–96) and domain II (residues 102–180) form a five-stranded antiparallel β-barrel structure with a chymotrypsin-like folding scaffold (20). The C-terminal domain III (residues 200–303) is a cluster of five α-helices linked to domain II by a long loop (residues 181–199). In the 3CLpro of SARS-CoV, domain III reportedly controls the dimerization and formation of the active enzyme (21).

The 3CLpro active site is located at the interface between domains I and II (22). In contrast to the Ser–His–Asp triad of chymotrypsin, SARS-CoV-2 3CLpro contains a catalytic His–Cys dyad in which the catalytic residue, Cys145, is located 2.5 Å from the carbonyl carbon of the conserved glutamine of the peptide substrate. The side chains of His41 and Cys145 of the catalytic dyad, which are part of domains I and II, respectively, form H-bond at 3.6 Å. The enzyme catalyzes the cleavage of the 11 nsps by targeting a highly conserved sequence comprising glutamine at P1 and leucine/phenylalanine/valine at P2 (*i.e.*, (L/F/V)Q↓(S, A or G), where ↓ defines the cleavage site) (12). Substrate binding to the active site of 3CLpro is highly specific, with a well-defined binding site consisting of four pockets (S1–S4) (23). Recent high-resolution crystal structures of 3CLpro complexed with nine substrate peptides and six cleavage products revealed a network of conserved hydrogen bonding interactions between the active-site residues of 3CLpro and the peptide substrate (24). Specifically, the side chain of the conserved glutamine at P1 of the peptide substrate forms hydrogen bonds with the side chains of His163 and Glu166 in the active site of 3CLpro and the backbone of Phe140. This network of hydrogen bonds is further stabilized by Asn142 (both side and main chains) and Ser1 from one monomer, which interact with Phe140 and Glu166 of the other monomer. The substrate-binding pocket in 3CLpro has 3 conserved histidine residues that affect enzyme activity depending on their protonation states, as demonstrated by molecular dynamics (MD) studies (Fig. 1) (25, 26, 27).

**Figure 1:**
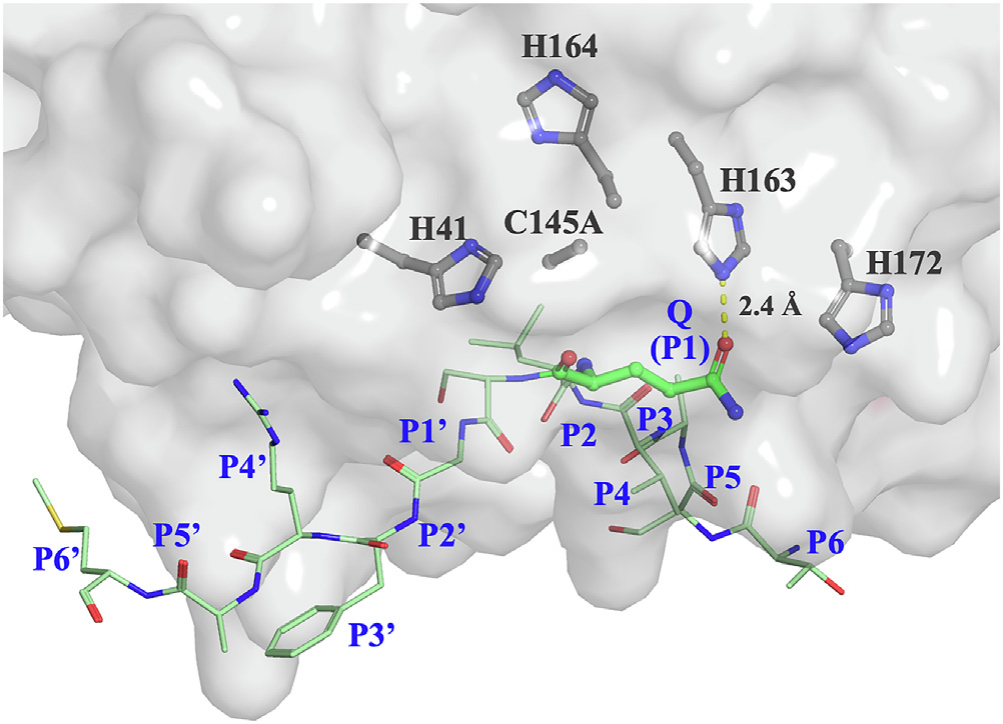
**Surface representation of the active site of SARS-CoV-2 3CLpro.** Protein Data Bank (ID: 7T70) was used to generate the figure. The catalytic residue Cys145 was mutated to Ala to prevent cleavage of the peptide substrate (24). The side chains of the catalytic dyad residues (His41 and Cys145) and the conserved active-site histidine residues (H163, H164, and H172) are depicted as *dark gray sticks*. The 12-mer substrate peptide (TSAVLQ↓SGFRKM) is colored in *green*. The conserved glutamine at P1 of the peptide substrate forms a hydrogen bond at 2.4 Å with the side chain of His163. The figure was generated using PyMol (Schrodinger LLC). 3CLpro, 3-chymotrypsin-like protease; SARS-CoV-2, severe acute respiratory syndrome coronavirus 2.

Crystallographic, biochemical, and MD studies have shown that the conformational flexibility and stability of 3CLpro are pH dependent. The potential effect of pH on SARS-CoV 3CLpro activity was first suggested by the significant differences in conformation between 3CLpro crystals grown at pH 6.0 and those grown at pH 7.6 or pH 8.0 (25). SARS-CoV 3CLpro exhibits a bell-shaped pH profile of proteolytic activity, with a peak at pH 7.0 to 7.4 (21, 25). However, no study has reported experimental pH profile data on SARS-CoV-2 3CLpro or examined the effects of the conserved histidine residues near the catalytic dyad on the pH dependence of its catalytic activity. Here, we performed site-directed mutagenesis to assess the effects of the conserved histidine residues in the substrate-binding sites (His163, His162, and His172) on the catalytic activity of SARS-COV-2 3CLpro. Importantly, elucidation of the pH profile of SARS-CoV-2 3CLpro enabled the proposal of a chemical mechanism for this protease.

## Results

### pH profile of WT SARS-CoV-2 3CLpro

**Figure 2:**
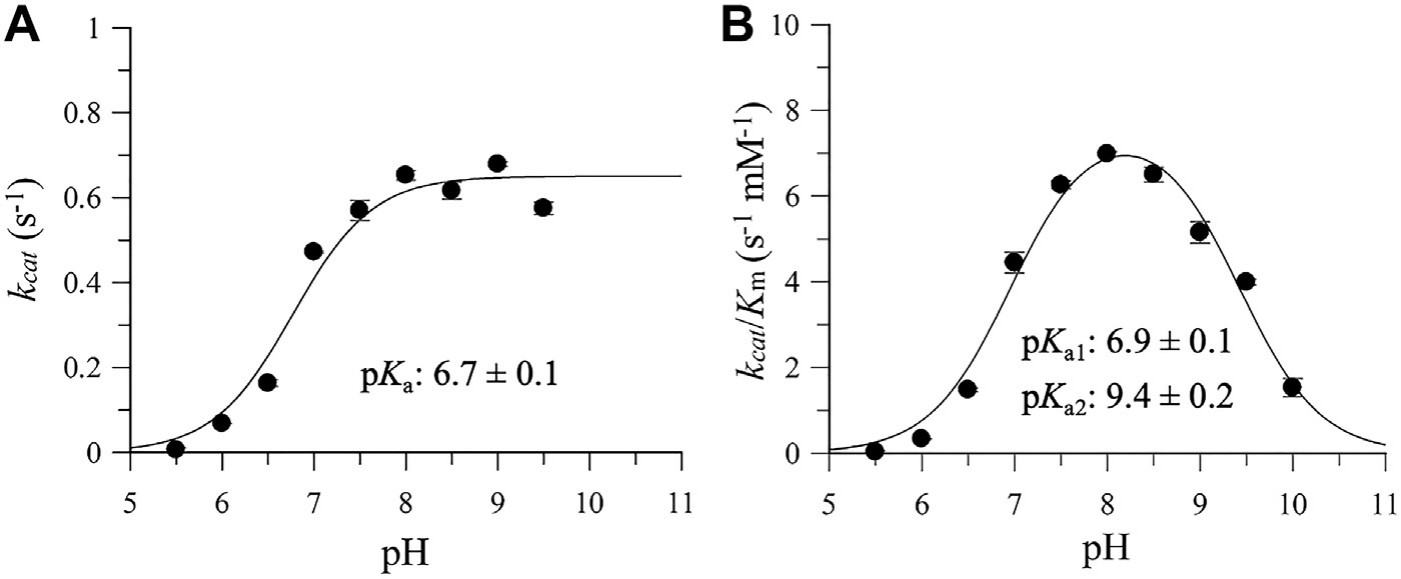
**pH profiles of WT SARS-CoV-2 3CLpro.***A*, pH profile of *k*_cat_ showing dependence on a single ionizable group with a p*K*_a_ of 6.7 ± 0.1. *B*, pH profile of *k*_cat_/*K*_*m*_ showing dependence on 2 ionizable groups with p*K*_a_ values of 6.9 ± 0.1 and 9.4 ± 0.2. The *lines* represent the best fit of the experimental data to half-bell (*k*_cat_) and bell-shaped (*k*_cat_/*K*_*m*_) models using GraFit 5.0 software (Erithacus Software Ltd). All reactions were performed at 30 °C with (DABCYL)KTSAVLQ↓SGFRKME(EDANS)-NH2 as the substrate. Data points are means ± SD of triplicate measurements. 3CLpro, 3-chymotrypsin-like protease; SARS-CoV-2, severe acute respiratory syndrome coronavirus 2.

The pH dependence of the kinetic parameters of WT 3CLpro from SARS-CoV-2, including the turnover number (*k*_cat_) and catalytic efficiency (*k*_cat_/*K*_*m*_), was determined over the pH range of 5.5 to 10.0 (Fig. 2). The proteolytic activity of WT 3CLpro was assayed continuously by monitoring the cleavage of the fluorescent peptide substrate using a highly sensitive FRET-based enzymatic assay (12, 22, 28, 29, 30). Initial velocity studies were performed at 30 °C in 20 mM Hepes (pH 7.0), 100 mM NaCl, 1 mM EDTA, 1 mM Tris(2-carboxyethyl)phosphine (TCEP), and 20% (v/v) dimethyl sulfoxide (DMSO) to acquire the kinetic parameters of the WT enzyme. The peptide substrate was varied from 20 to 500 μM at a fixed enzyme concentration, and the proteolytic cleavage rate was fit to Michaelis–Menten equation.

The pH profile of *k*_cat_ had a half-bell shape with maximum *k*_cat_ values at pH >7.5 as shown in Figure 2*A*. Fitting the *k*_cat_ pH profile using Equation 1 resulted in a single p*K*_a_ value of 6.7 ± 0.1. By contrast, the pH profile of *k*_cat_/*K*_*m*_ was bell shaped, with a maximum *k*_cat_/*K*_*m*_ value at pH 8.0 (Fig. 2*B*). Fitting the data to a double-titration bell-shaped model (Equation 2) resulted in p*K*_a_ values of 6.9 ± 0.1 and 9.4 ± 0.1. Despite similar *k*_cat_ values, *k*_cat_/*K*_*m*_ values were lower at pH 9.0 to 9.5 than at pH 7.5 to 8.5. This observation is due to an increase in *K*_*m*_ values (Table S1), which implies that substrate binding is reduced at high pH. Experimentally, we also observed aggregation and precipitation of the peptide substrate at pH ≥10.

### Enzymatic activity and initial velocity studies of histidine mutants of SARS-CoV-2 3CLpro

**Figure 3:**
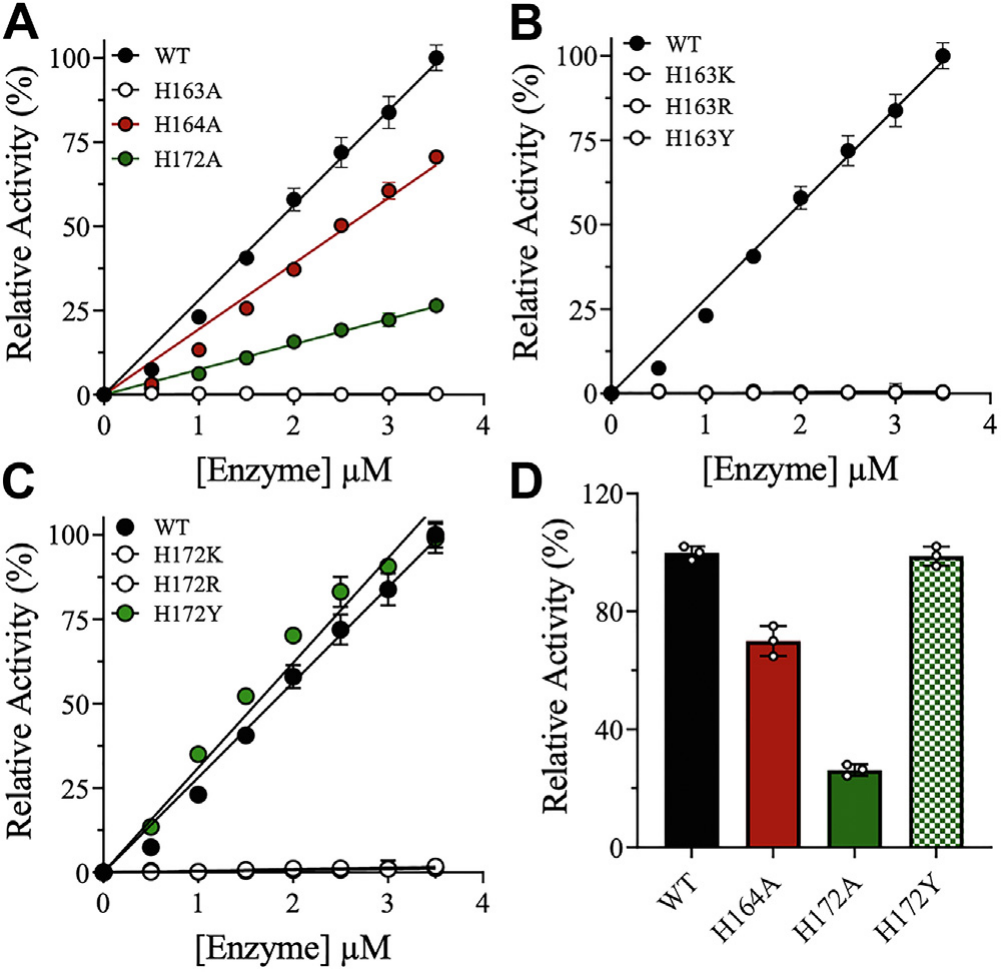
**Effects of 3CLpro histidine (H163, H164, and H172) mutants on activity relative to WT.***A*–*C*, relative activities of the active-site histidine mutants of 3CLpro at increasing enzyme concentrations (0.0–3.5 μM) and a fixed peptide substrate concentration of 60 μM. The proteolytic cleavage rates of each mutant were normalized to the rate of 3CLpro WT to obtain the percent relative enzymatic activity. Enzymatically active mutants are represented by *filled colored circles*, whereas enzymatically inactive mutants are represented by *open black circles*. *D*, bar plot of the relative activity of WT 3CLpro and the enzymatically active mutants (H164A, H172A, and H172Y). Data are presented as the mean ± SD, n = 3. 3CLpro, 3-chymotrypsin-like protease.

Crystal structure analysis revealed network of bonding interactions with the peptide substrate in the active site of 3CLpro including 3 histidine residues (Fig. 1). To determine the roles of the conserved histidine residues in the substrate-binding site of SARS-CoV-2 3CLpro, we introduced alanine mutations at H163, H164, and H172. The proteolytic activity of the histidine mutants was compared with the WT 3CLpro, where the rate was measured at a fixed peptide substrate concentration of 60 μM, whereas the enzyme concentration was varied from 0.5 to 5.0 μM (Fig. 3). Increasing the enzyme concentration was important for activity detection, as the mutants were expected to have low activity.

Alanine substitution at His163 (H163A) completely inactivated the enzymatic activity of 3CLpro (Fig. 3*A*). By contrast, H164A and H172A retained 75% and 26% of the enzymatic activity of WT 3CLpro, respectively (Fig. 3*D*). These results suggested that H163 and H172 are important for maintaining the catalytic activity of SARS-CoV-2 3CLpro. In addition to alanine substitutions, we introduced lysine, arginine, and tyrosine at H163 and H172. None of the amino acid substitutions of His163, that is, H163K, H163R, and H163Y, recovered the enzymatic activity of 3CLpro, further supporting the importance of His163 in the catalytic mechanism of 3CLpro (Fig. 3*B*). However, tyrosine substitution at His172 resulted in full recovery of enzymatic activity to WT levels (Fig. 3, *C* and *D*).

**Figure 4:**
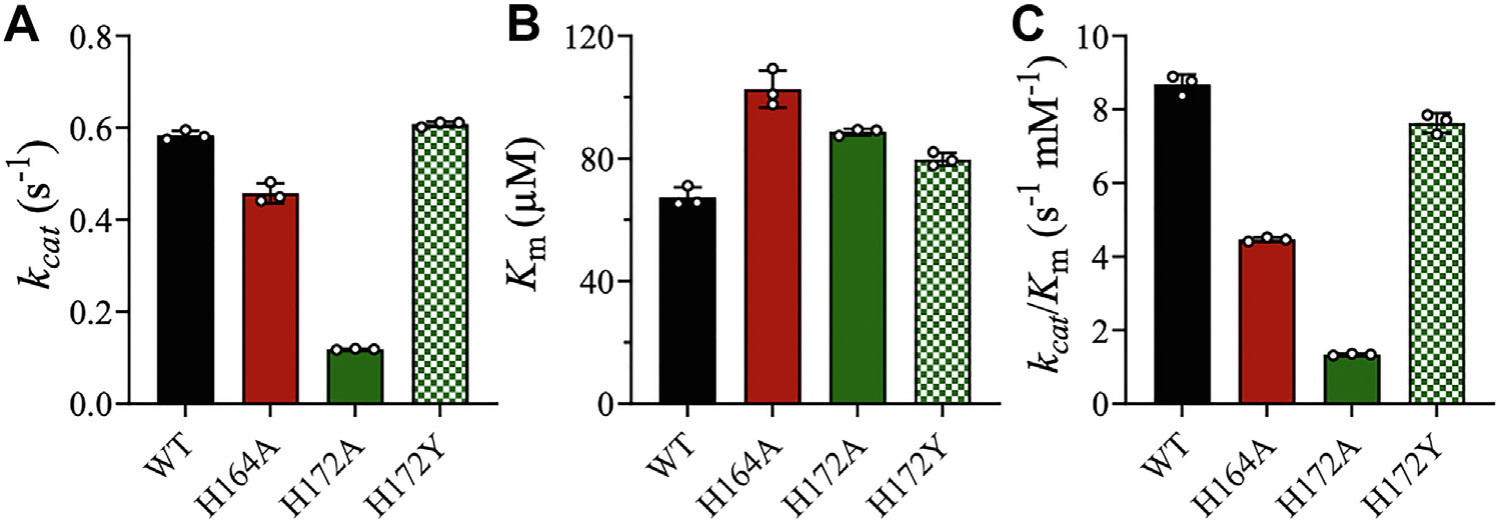
**Effects of 3CLpro histidine mutants on the kinetic parameters.** (*A*) *k*_cat_, (*B*) *K*_*m*_, and (*C*) *k*_cat_/*K*_*m*_ of 3CLpro compared with WT enzyme. Data points are the means ± SD of triplicate measurements. 3CLpro, 3-chymotrypsin-like protease.

Next, initial velocity studies were performed to acquire the kinetic parameters of the H164A, H172A, and H172Y mutants, which had partial or full catalytic activity compared with the WT enzyme (Fig. 4). Compared with WT 3CLpro, H164A and H172A exhibited 22% and 80% reductions in *k*_cat_, respectively, whereas the *k*_cat_ of H172Y was nearly identical to the WT enzyme (Fig. 4*A*). All tested mutations increased the *K*_*m*_ of 3CLpro compared with WT, which had a *K*_*m*_ of 67 ± 3 mM; the *K*_*m*_ values of H164A, H172A, and H172Y were 103 ± 6 mM, 89 ± 1 mM, and 80 ± 2 mM, respectively (Fig. 4*B*). These effects indicate that the histidine residues play important roles in peptide substrate binding. Overall, the WT and H172Y enzymes had the highest and similar catalytic efficiencies (Fig. 4*C*), whereas H164A and H172A exhibited decreases in *k*_cat_/*K*_*m*_ of 49% and 85%, respectively.

### Thermodynamic stability of histidine mutants of SARS-CoV-2 3CLpro

**Figure 5:**
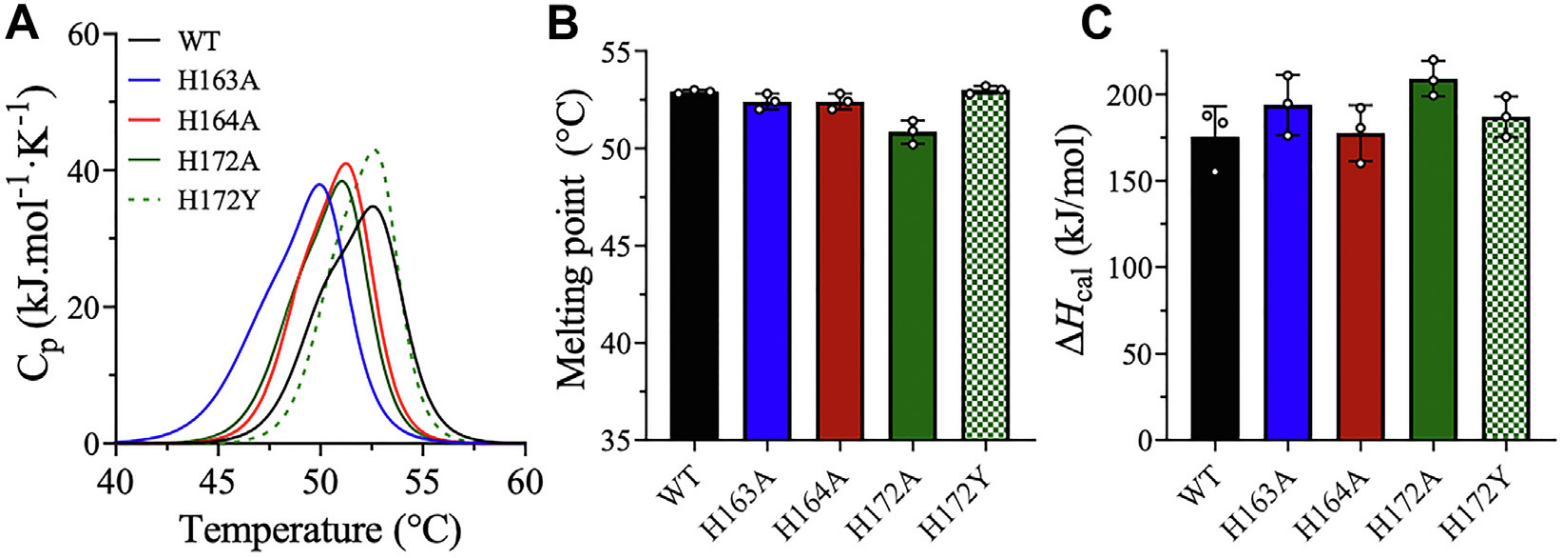
**DSC thermal scans of WT and mutant 3CLpro**. *A*, DSC thermal scans of WT and mutant 3CLpro (H163A, H164A, H172A, and H172Y) were acquired at a heating rate of 1.0 °C/min. *B*, bar plot of *T*_m_ calculated at the apex of the thermographic peak. *C*, bar plot of Δ*H*_cal_ calculated from the area under the DSC thermal peak. Data are presented as the mean ± SD, n = 3. 3CLpro, 3-chymotrypsin-like protease; DSC, differential scanning calorimetry.

The effects of the histidine mutations (H163A, H164A, H172A, and H172Y) on the thermodynamic stability of 3CLpro were examined using differential scanning calorimetry (DSC). DSC thermograms of each enzyme were acquired in 20 mM Hepes (pH 7.4), 100 mM NaCl, and 0.5 mM TCEP, and the temperature was ramped from 15 to 75 °C at a scan rate of 1 °C/min to acquire the thermal unfolding transitions (Fig. 5). The WT and mutant enzymes exhibited a single transition with an early shoulder peak (Fig. 5*A*). The melting temperature (*T*_m_) was determined from the apex of the thermogram peak, where the WT enzyme had a *T*_m_ of 52.9 ± 0.1 °C, consistent with previously reported values (22, 31). The alanine substitutions at H163 and H172 decreased the *T*_m_ slightly to 50.3 ± 0.1 °C and 50.8 ± 0.6 °C, respectively, whereas H172Y had a *T*_m_ of 53.0 ± 0.2 °C, similar to the WT enzyme (Fig. 5*B*). The calorimetric enthalpy (Δ*H*_cal_) values determined from the area under the thermographic peak were similar for all variants: 185 ± 3 kJ/mol for WT, 194 ± 17 kJ/mol for H163A, 186 ± 8 kJ/mol for H164A, 209 ± 10 kJ/mol for H172A, and 187 ± 16 kJ/mol for H172Y (Fig. 5*C*).

### pH profiles of the histidine mutants of SARS-CoV-2 3CLpro

**Figure 6:**
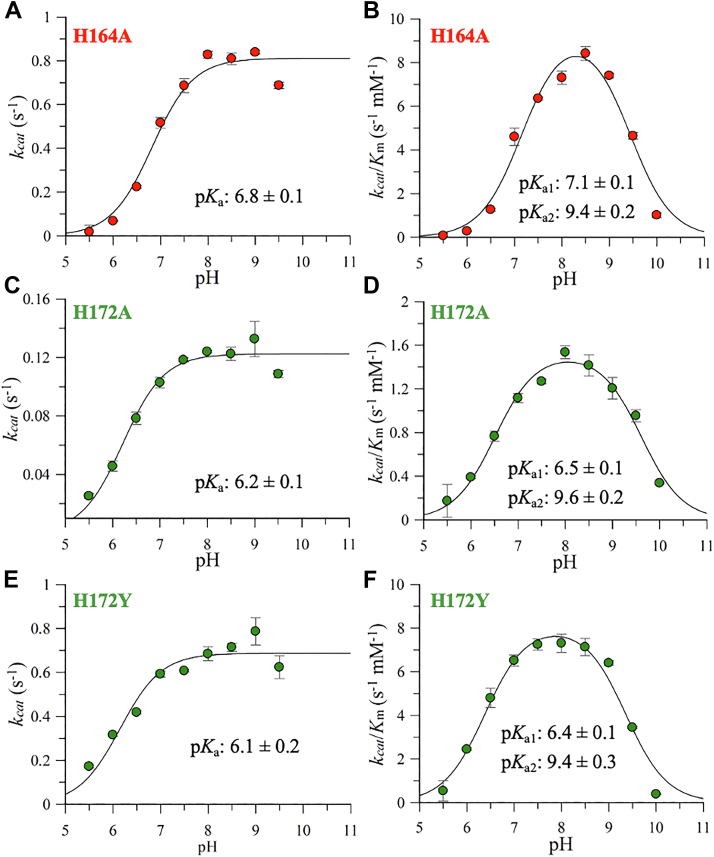
**pH profiles of SARS-CoV-2 3CLpro histidine mutants.***A*, *C*, and *E*, pH profiles of *k*_cat_ for H164A, H172A, and H172Y showing dependence on a single ionizable group with one p*K*_a_ value. *B*, *D*, and *F*, pH profiles of *k*_cat_/*K*_*m*_ for H164A, H172A, and H172Y showing dependence on 2 ionizable groups with 2 p*K*_a_ values. The *lines* represent the best fit of the experimental data to half-bell (*k*_cat_) and bell-shaped (*k*_cat_/*K*_*m*_) models using GraFit 5.0 software (Erithacus Software Ltd). Data points are means ± SD of triplicate measurements. 3CLpro, 3-chymotrypsin-like protease; SARS-CoV-2, severe acute respiratory syndrome coronavirus 2.

Given its complete loss of enzymatic activity, the pH profiles of H163 mutants of 3CLpro could not be determined. The pH profiles of the catalytically active histidine mutants H164A, H172A, and H172Y were similar to those of the WT enzyme (Fig. 6). The *k*_cat_ pH profiles exhibited a half-bell shape with one ionizable residue with a p*K*_a_ value of 6.8 ± 0.1, 6.2 ± 0.1, or 6.1 ± 0.2 for H164A, H172A, and H172Y, respectively (Fig. 6, *A*, *C* and *E*). The *k*_cat_/*K*_*m*_ pH profiles exhibited a bell shape with 2 ionizable groups with p*K*_a_ values similar to those of the WT enzyme (6.9 ± 0.1 and 9.4 ± 0.2): H164A (7.1 ± 0.1 and 9.4 ± 0.2), H172A (6.5 ± 0.1 and 9.6 ± 0.2), and H172Y (6.4 ± 0.1 and 9.4 ± 0.3) (Fig. 6, *B*, *D* and *F*). For H172A and H172Y, the first p*K*_a_ was slightly lower than that of WT. Importantly, the overall kinetic parameters of H172A were lower than those of WT at all tested pH values.

## Discussion

The cysteine protease 3CLpro is highly conserved in all coronaviruses because of its essential role in processing viral polyproteins (5, 8, 9, 10). MD and crystallographic studies have indicated that coronavirus 3CLpro enzymes undergo pH-dependent conformational changes (23, 25, 32). This conformational dependence on pH is due to the high flexibility of 3CLpro enzymes and is physiologically relevant: 3CLpro is assembled in late endosomes, where the low pH environment maintains the enzyme in an inactive state to prevent autoproteolysis of the viral polyproteins (33). Crystal structures of SARS-CoV 3CLpro have been determined in different pH environments (34). The dimeric structures of 3CLpro at pH 7.6 and 8.0 are in the fully active conformations, whereas at pH 6.0, 3CLpro undergoes substantial conformational changes that lead to complete inactivation of one protomer. These initial crystallographic data were later supported by several pH profiles of SARS-CoV 3CLpro, which revealed a bell-shaped curve with maximum activity at pH 7.0 to 8.5 (21, 25, 34).

In the present study, we identified key residues in the active site of SARS-CoV-2 3CLpro that interact with the substrate, in addition to the catalytic dyad His41 and Cys145. Among these residues, His163, His164, and His172 play important roles in binding the peptide substrate and ensuring its proper orientation for catalysis. Within the peptide substrate, the Gln residue has the largest number of binding interactions with the protease. The S1 pocket of the active site of 3CLpro is mainly formed by residues Phe140, Leu141, Asn142, Glu166, His163, and His172 from one monomer and Ser1 of the other monomer (35). The ionizable groups in the active site of an enzyme must adopt the proper orientation to ensure substrate binding and functional catalysis. Although previous MD and crystallographic studies have discussed the pH-dependent conformational changes of 3CLpro of SARS-CoV and SARS-CoV-2, this study is the first to report experimental pH profiles of the kinetic parameters of the WT enzyme and variants with mutations of key histidine residues at the substrate-binding site of SARS-CoV-2 3CLpro.

The pH profile of *k*_cat_/*K*_*m*_ of SARS-CoV-2 3CLpro is bell shaped with p*K*_a_ values of 6.9 ± 0.1 and 9.4 ± 0.1 (Fig. 2), which are likely attributable to the ionizable side chains of the catalytic dyad His41 and Cys145, respectively. The pH profiles of *k*_cat_ and *k*_cat_/*K*_*m*_ of H164A, H172A, and H172Y are similar in shape to those of the WT enzyme, with comparable p*K*_a_ values (Fig. 6). The similar shapes of the pH profiles and calculated p*K*_a_ values of the mutants and the WT enzyme further indicate that the estimated p*K*_a_ values correspond to the catalytic dyad His41 and Cys145. Our findings are in agreement with previous MD studies proposing a general base mechanism of 3CLpro in which the catalytic residues are neutral at physiological pH (*i.e.*, non–ion-pair mechanism), with p*K*_a_ values of 6.4 and 8.3 for the catalytic dyad His41 and Cys145, respectively (21, 26, 27, 35, 36, 37). A comprehensive MD study of the neutral and zwitterionic states of the catalytic dyad of SARS-CoV-2 3CLpro found enhanced binding and stability of the peptide substrate in the proper mode for catalysis when the catalytic residues were in the neutral form (27). By contrast, the zwitterionic state of 3CLpro disturbed domain I of the active site and impaired substrate binding (27). Our experimental pH profiles of *k*_cat_/*K*_*m*_ support the need for a neutral state of the catalytic dyad to ensure proper substrate binding (*i.e.*, low *K*_*m*_ values) and maximal enzyme catalysis (high *k*_cat_ values). However, the pH profile of *k*_cat_ yielded only one titratable group, the imidazole side chain of His41, needed for optimal enzyme catalysis, with a p*K*_a_ of 6.7 ± 0.1.

In addition to the active-site residues, computational studies of the protonation states of conserved histidine residues in the substrate-binding site have reported that H163 and H172 have p*K*_a_ values of <5.0 and 6.6, respectively, and thus exist in their neutral states under physiological conditions (26, 36). At physiological pH, the neutral (singly protonated Nε) state of His163 facilitates polar and nonpolar contacts to maintain a stable S1-binding pocket. In fact, a structural analysis of the 3CLpro substrate-binding site found that an H-bond forms between His163 (at Nε) and the highly conserved Gln substrate residue (at its side-chain carbonyl oxygen); this interaction requires a neutral His163 to ensure the absolute specificity of 3CLpro for Gln at P1 of the peptide substrate (34).

Our work demonstrates that hydrogen bonding interactions between His163 (at Nε) with the side-chain carbonyl oxygen of P1 Gln of the peptide substrate at a 2.4 Å is crucial for the activity of 3CLpro of SARS-CoV-2 (Fig. 1). Hence, different amino acid substitutions of H163 including substitution of arginine (H163R), lysine (H164K), and tyrosine (H163Y) could not recover the activity of 3CLpro. Even though, arginine, lysine, and tyrosine are able to form hydrogen bonding interaction; however, the large size of their side chains did not facilitate the required H-bonding distances with the peptide substrate. Overall, H-bond with specific distance between His163 and P1 of the peptide substrate is crucial to facilitate proper peptide substrate orientation for optimum activities of 3CLpro of SARS-CoV-2.

In addition, His163 participates in aromatic stacking with Phe140 to support the neutral state of His163 and ensure its optimal interaction with the substrate Gln (26, 34). The neutral side chain of His163 (at Nδ) also acts as an H-bond acceptor in an H-bond interaction with the side chain of the donor Tyr161 (38). MD simulations further revealed that full protonation of H163 results in the spontaneous collapse of the binding pocket and inactivation of 3CLpro (25, 38).

The estimated p*K*_a_ of H172 was 6.6; thus, this residue is also neutral in the optimum pH range (7.5–8.5) of WT 3CLpro (26, 36). In fact, MD simulations have indicated that protonation of His172 at pH 6.0 results in collapse of the oxyanion hole, leading to conformational deactivation of the S1 pocket (23, 26). The imidazole side chain of the neutral H172 forms a conserved H-bond with the side chain of Glu166, a key interaction that is lost upon H172 protonation (19, 25, 34). In addition, a computational analysis demonstrated that protonation of His172 abolishes its interaction with Ser1 in the N-finger domain of the opposite monomer, whereas this interaction is maintained when H172 is in the neutral state (26).

Consequently, our results provide experimental evidence of the important roles of H163 and H172 in maintaining the catalytic activity of 3CLpro. Alanine substitution of H163 resulted in complete loss of enzymatic activity, and substitution with other amino acids did not recover 3CLpro activity (Fig. 3, *A* and *B*). Our data provide experimental proof of the reported interaction between the imidazole of His163 (at Nε2) and the highly conserved Gln residue of the peptide substrate (38). Alanine substitution of H172 reduced the catalytic activity and kinetic parameters of 3CLpro by 80% compared with WT (Figure 3, Figure 4). These reductions may be due to the loss of the H-bond interaction between the side chains of His172 and Glu166, which was previously reported to be critical for stabilizing the oxyanion hole and hence activating enzyme function (19, 23). We experimentally verified the neutral state of His172 by introducing lysine, arginine, and tyrosine mutations and assessing the activity and pH profiles of the resulting mutants (Figure 3, Figure 4, Figure 6). H172K and H172R were inactive, and only H172Y resulted in full recovery of activity similar to that of WT 3CLpro (Fig. 3*D*).

**Figure 7:**
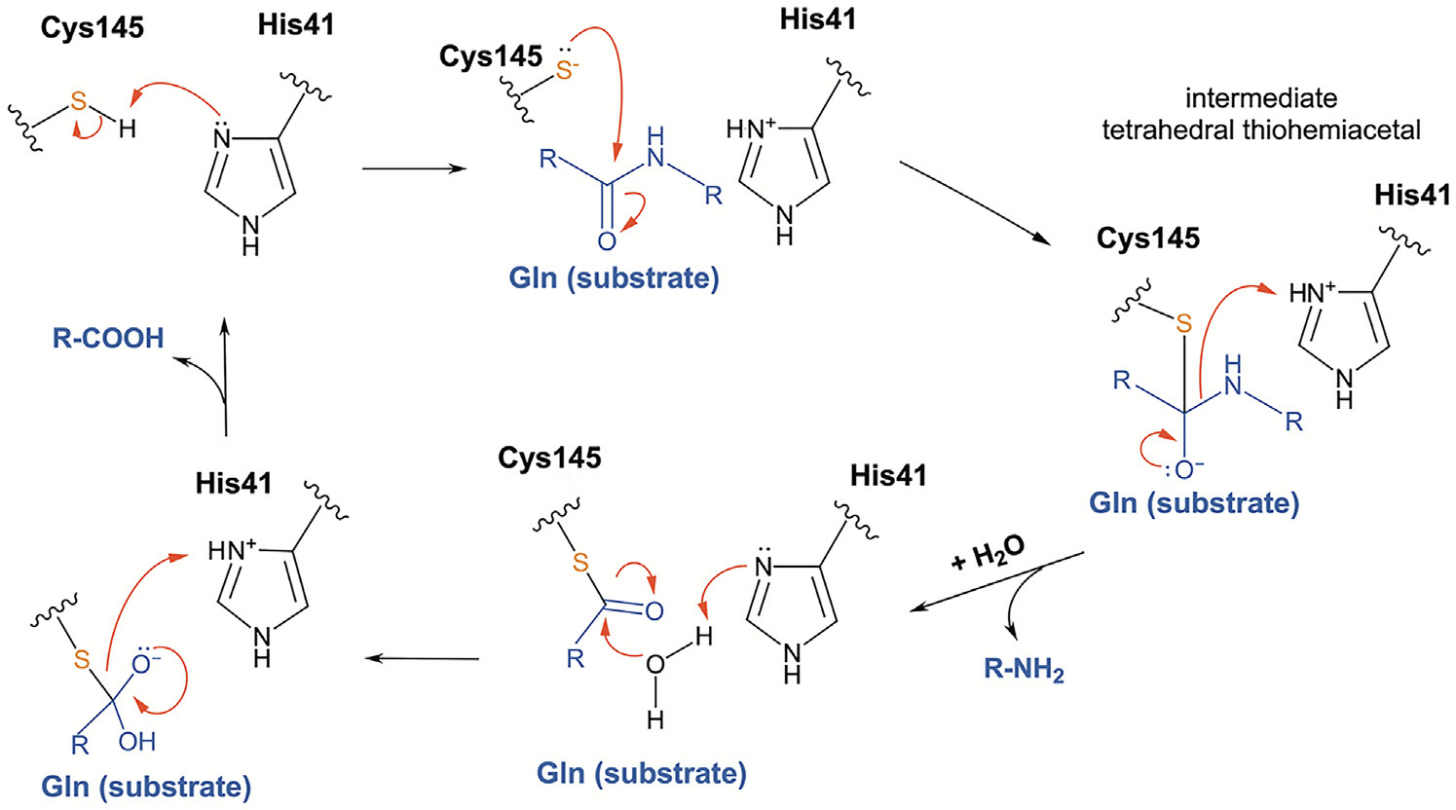
**Proposed chemical mechanism of 3CLpro SARS-CoV-2**. 3CLpro, 3-chymotrypsin-like protease; SARS-CoV-2, severe acute respiratory syndrome coronavirus 2.

In light of these findings, a catalytic mechanism of 3CLpro can be proposed (Fig. 7). Catalysis begins with the deprotonation of the thiol side chain of Cys145 by His41 to facilitate nucleophilic attack of Cys145 on the carbonyl carbon of glutamine in the polyprotein backbone and the formation of a covalent thioester bond. The resulting tetrahedral thiohemiacetal intermediate contains an oxyanion group that is stabilized by hydrogen bonding with the amides of the main-chain residues Ser139–Leu141. Subsequent collapse of the thiohemiacetal complex releases the C-terminal segment of the polypeptide substrate (20, 21, 39), and hydrolysis of the thioester linkage by a water molecule displaces Cys145 and releases the N-terminal part of the polypeptide substrate.

In summary, our experimental data and previous MD studies support a general base mechanism of SARS-CoV-2 3CLpro in which the neutral states of the catalytic dyad residues and conserved active-site histidine residues are required for catalysis. In addition, we highlight the importance of the neutral states of His163 and His172 for achieving a fully active enzyme state.

## Experimental procedures

### Expression and purification of WT and mutant 3CLpro

The recombinant genes for WT and mutant 3CLpro were introduced into pET28b(+) bacterial expression vectors by GenScript, Inc. The vectors were used to transform *Escherichia coli* BL21-CodonPlus-RIL (Stratagene) for protein expression as previously described (31). The inoculated culture (1 l) was grown in terrific broth medium at 30 °C in the presence of 100 mg/l kanamycin and 50 mg/l chloramphenicol until the absorbance at 600 nm reached 0.8. The temperature was then lowered to 15 °C, and protein expression was induced overnight with 0.5 mM IPTG. The cells were harvested by centrifugation at 8000 rpm and 4 °C for 10 min in an Avanti J26-XPI centrifuge (Beckman Coulter, Inc). The cells were homogenized in lysis buffer containing 20 mM Tris (pH 7.5), 150 mM NaCl, 5 mM imidazole, 3 mM β-mercaptoethanol (β-ME), and 0.1% protease inhibitor cocktail (Sigma–Aldrich; catalog no.: P8849). The cell lysates were sonicated on ice before centrifugation at 40,000*g* for 45 min at 4 °C.

The supernatant was loaded onto a ProBond nickel-chelating column (Life Technologies) previously equilibrated with binding buffer containing 20 mM Tris (pH 7.5), 150 mM NaCl, 5 mM imidazole, and 3 mM β-ME at 4 °C. The column was washed with binding buffer followed by washing buffer containing 20 mM Tris (pH 7.5), 150 mM NaCl, 25 mM imidazole, and 3 mM β-ME. The Hisx6-tagged 3CLpro enzyme was eluted with 20 mM Tris (pH 7.5), 150 mM NaCl, 300 mM imidazole, and 3 mM βME. Finally, the fractions containing 3CLpro were loaded onto a HiLoad 16/600 Superdex 200 size-exclusion column (GE Healthcare) on an ÄKTA pure 25 chromatography system (Cytiva). The gel filtration column was pre-equilibrated with 20 mM Hepes (pH 7.5), 100 mM NaCl, and 0.5 mM TCEP. The final protein was collected and concentrated to 55 μM as determined by the Bradford assay, and the protein purity was assessed *via* SDS-PAGE.

### Enzymatic activity analysis

The enzymatic activities of WT 3CLpro and the histidine mutants were assessed by an FRET-based assay using the 14-amino-acid fluorogenic peptide substrate (DABCYL)KTSAVLQ↓SGFRKME(EDANS)-NH_2_ (GenScript, Inc) as described previously (12, 19, 28, 29, 40, 41, 42). The reaction was initiated by adding WT or mutant 3CLpro to the peptide substrate in 20 mM Hepes, pH 7.0, 100 mM NaCl, 1 mM EDTA, and 1 mM TCEP. The assay buffer contained 20% (v/v) DMSO to reduce the aggregation of the peptide substrate and enhance its stability (22). The reaction rate was measured for 10 min at 30 °C in a thermostatically controlled cell compartment. The catalytic rates were determined from the cleavage of the fluorogenic substrate, which was monitored by the increase in the fluorescence signal upon release of the EDANS group in a 96-well plate assay format in a Cytation 5 multimode microplate reader (Biotek Instruments). The fluorescence signal was monitored at λ_excitation_ of 360 nm and λ_emission_ of 500 nm.

To account for the inner filter effect in the FRET enzymatic assay, first, the excitation coefficient of free EDANS was determined in the absence of the peptide substrate by varying the concentration of free EDANS, *f*_0_ (EDANS). Next, the correction factor (Corr%) required to correct for the decrease in the emission signal of the fluorogenic substrate in the presence of the quencher (DABCYL) was calculated (22, 40, 41, 43, 44). To calculate Corr%, the fluorescence of a fixed concentration (50 μM) of free EDANS was measured in the absence, f(S), and presence, f(S + EDANS), of various concentrations of the peptide substrate (from 20 to 500 μM):

$$f_{s}\;\left(\text{EDANS}\right)=f\left(S+\text{EDANS}\right)-f\left(S\right)$$

To determine Corr%, the emission reduction of free EDANS at a specific substrate concentration, *f*_s_ (EDANS), was compared with that of EDANS in the absence of peptide substrate, *f*_o_ (EDANS).

$$\text{Corr}=\frac{f_{s}\;\left(\text{EDANS}\right)}{f_{0}\;\left(\text{EDANS}\right)}$$

The values of Corr% calculated at different peptide substrate concentrations were taken into consideration when measuring the cleavage rate of 3CLpro. The effect of histidine mutations on the catalytic rate of 3CLpro was determined by measuring enzymatic activity at different enzyme concentrations ranging from 0.5 to 5.0 μM and a fixed peptide substrate concentration of 60 μM. The relative activity of each mutant was obtained from the slope of the straight line for each mutant.

### Initial velocity studies and pH dependence of kinetic parameters

Next, initial velocity studies were performed to determine the kinetic parameters *k*_cat_ and *K*_*m*_ for the WT enzyme and the histidines' catalytically active histidine mutants. The concentration of the peptide substrate was varied from 20 to 500 μM at a fixed enzyme concentration. Three independent experiments were performed with triplicate measurement each to obtain the cleavage rate data that were fitted to the Michaelis–Menten equation using the global fitting analysis function in the kinetics module of SigmaPlot (Systat Software, Inc). The kinetic parameters, *k*_cat_ and *K*_*m*_, were obtained, and the standard error bars were calculated from triplicate measurements of each reaction using GraphPad Prism 9.0 software (GraphPad Software, Inc). The results are presented as the mean ± SD.

The pH profiles of WT 3CLpro and the catalytically active mutants H164A, H172A, and H172Y were measured using the FRET enzymatic assay by varying the concentration of the peptide substrate from 20 to 500 μM at a fixed enzyme concentration. The reaction buffers were prepared over a pH range of 5.5 to 10 using 20 mM Mes for pH 5.5 to 6.5, 20 mM Hepes for pH 7.0 to 8.0, and 20 mM Ches for pH 8.5 to 10.0. All reaction buffers contained 100 mM NaCl, 1 mM EDTA, 1 mM TCEP, and 20% (v/v) DMSO. Initial velocity studies were performed to determine the kinetic parameters *k*_cat_ and *k*_cat_/*K*_*m*_ as a function of pH. GraFit 5.0 software (Erithacus Software Ltd) was used to determine the p*K*_a_ values. Equation 1 was used to fit the pH profile data of *k*_cat_ with a single ionizable group resulting in a half-bell curve with zero activity at low pH and an activity plateau at high pH. Equation 2 was used to fit the pH profile data of *k*_cat_/*K*_*m*_ with 2 ionizable groups resulting in a bell-shaped curve with zero activity at low and high pH.

(1) $$k=k_{\left(limit\right)}\frac{10^{pH-pKa}\;}{10^{pH-pKa}+1}$$

(2) $$k=k_{\left(limit\right)}\left[\frac{1}{1+10^{pK1-pH}+10^{pH-pK2}}\right]$$

In Equation 1, *k* is *k*_cat_, *k*_(limit)_ corresponds to the maximum limit of *k*_cat_, and p*K*_a_ is the dissociation constant of the single ionizable group. In Equation 2, *k* is *k*_cat_/*K*_*m*_, *k*_(limit)_ corresponds to the maximum limit of *k*_cat_/*K*_*m*_, and p*K*_a1_ and p*K*_a2_ are the dissociation constants of the first and second ionizable groups. The 3 independent pH profile measurements were analyzed using GraFit 5.0 software that provided the p*K*_a_ values and standard errors.

### DSC

The effects of mutations on the thermodynamic stability of 3CLpro were assessed by DSC in a Nano-DSC instrument (TA Instruments). A fixed enzyme concentration of 25 μM was used in buffer containing 20 mM Hepes (pH 7.4), 100 mM NaCl, and 0.5 mM TCEP. All samples were scanned from 15 to 75 °C at a temperature ramp rate of 1 °C/min. The buffer was used as a reference, and the protein samples were degassed for 10 min prior to the start of each analysis run. The DSC scans were acquired by ramping up the temperature twice to obtain 2 thermograms; the second scan was used as the buffer background for each sample. The lack of signal in the second ramp-up temperature scan confirmed that the melting transitions of all 3CLpro variants were irreversible. The DSC scans were normalized for protein concentration and baseline corrected by subtracting the corresponding buffer baseline. The data were then converted to plots of excess heat capacity (C_p_) as a function of temperature. The *T*_m_ of 3CLpro was determined from the temperature at the apex of the thermal transition, and the calorimetric enthalpy (Δ*H*_cal_) of the transition was estimated from the area under the thermal transition curve using NanoAnalyze Software, version 3.11.0 (TA Instruments).

## Data availability

The authors declare that all data that support the findings of this study are available within this article and its accompanying files.

## Supporting information

This article contains supporting information.

## Conflict of interest

The authors declare that they have no conflicts of interest with the contents of this article.
