# Supplementary material for: pH profiles of 3-chymotrypsin-like protease (3CLpro) from SARS-CoV-2 elucidate its catalytic mechanism and a histidine residue critical for activity
Source: J Biol Chem. 2022 Dec 9;299(2):102790. doi: 10.1016/j.jbc.2022.102790 (PMC9733303; doi:10.1016/j.jbc.2022.102790)
Supplement: Supporting Information [file mmc1.docx]

**Supporting Information**

**pH profiles of 3-chymotrypsin-like protease (3CLpro) from SARS-CoV-2 elucidate its catalytic mechanism and a histidine residue critical for activity**

Kenana Al Adem^1†^, Juliana C. Ferreira^1†^, Samar Fadl^1^, and Wael M. Rabeh^1,*^

^1^Science Division, New York University Abu Dhabi, PO Box 129188, Abu Dhabi, United Arab Emirates.

^*^Corresponding author: wael.rabeh@nyu.edu

^†^These authors contributed equally to this work.

List of material included:

Table 1S: Kinetic parameters obtained from the pH profile experiments.

**Supplementary Table S1.**

Kinetic parameters (k_cat_ (s^-1^), K_m_ (mM) and k_cat_/K_m_ (s^-1^. mM^-1^) obtained from the pH profile experiments.

|  | **Wild-Type** | | |  |
| --- | --- | --- | --- | --- |
| **pH** | ***k*_cat_ (s^-1^)** | ***K*_m_ (mM)** | ***k*_cat_/*K*_m_ (s^-1^. mM^-1^)** | |
| 5.5 | 0.006 ± 0.001 | 0.189 ± 0.084 | 0.03 ± 0.003 | |
| 6 | 0.068 ± 0.001 | 0.203 ± 0.01 | 0.33 ± 0.01 | |
| 6.5 | 0.163 ± 0.007 | 0.110 ± 0.007 | 1.47 ± 0.04 | |
| 7 | 0.472 ± 0.002 | 0.106 ± 0.006 | 4.44 ± 0.24 | |
| 7.5 | 0.551 ± 0.023 | 0.081 ± 0.012 | 6.79 ± 0.1 | |
| 8 | 0.621 ± 0.011 | 0.085 ± 0.002 | 7.12 ± 0.1 | |
| 8.5 | 0.616 ± 0.019 | 0.090 ± 0.005 | 6.71 ± 0.2 | |
| 9 | 0.611 ± 0.005 | 0.131 ± 0.006 | 5.15 ± 0.25 | |
| 9.5 | 0.574 ± 0.014 | 0.144 ± 0.005 | 3.99 ± 0.06 | |
| 10 | 0.093 ± 0.011 | 0.062 ± 0.017 | 1.52 ± 0.21 | |

|  | **H164A** | | |
| --- | --- | --- | --- |
| **pH** | ***k*_cat_ (s^-1^)** | ***K*_m_ (mM)** | ***k*_cat_/*K*_m_ (s^-1^. mM^-1^)** |
| 5.5 | 0.017 ± 0.03 | 0.239 ± 0.001 | 0.07 ± 0.13 |
| 6 | 0.067 ± 0.001 | 0.242 ± 0.006 | 0.28 ± 0.01 |
| 6.5 | 0.223 ± 0.006 | 0.176 ± 0.014 | 1.26 ± 0.06 |
| 7 | 0.516 ± 0.025 | 0.113 ± 0.016 | 4.59 ± 0.40 |
| 7.5 | 0.686 ± 0.032 | 0.107 ± 0.006 | 6.35 ± 0.08 |
| 8 | 0.827 ± 0.016 | 0.113 ± 0.007 | 7.30 ± 0.31 |
| 8.5 | 0.809 ± 0.026 | 0.096 ± 0.006 | 8.41 ± 0.32 |
| 9 | 0.838 ± 0.008 | 0.113 ± 0.003 | 7.39 ± 0.10 |
| 9.5 | 0.687 ± 0.014 | 0.148 ± 0.004 | 4.64 ± 0.16 |
| 10 | 0.127 ± 0.026 | 0.129 ± 0.04 | 1.00 ± 0.11 |

|  | **H172A** | | |
| --- | --- | --- | --- |
| **pH** | ***k*_cat_ (s^-1^)** | ***K*_m_ (mM)** | ***k*_cat_/*K*_m_ (s^-1^. mM^-1^)** |
| 5.5 | 0.025 ± 0.001 | 0.097 ± 0.008 | 0.173 ± 0.15 |
| 6 | 0.045 ± 0.003 | 0.117 ± 0.011 | 0.391 ± 0.01 |
| 6.5 | 0.078 ± 0.004 | 0.103 ± 0.013 | 0.766 ± 0.05 |
| 7 | 0.102 ± 0.003 | 0.092 ± 0.005 | 1.116 ± 0.04 |
| 7.5 | 0.118 ± 0.001 | 0.093 ± 0.001 | 1.268 ± 0.02 |
| 8 | 0.124 ± 0.001 | 0.081 ± 0.003 | 1.535 ± 0.06 |
| 8.5 | 0.122 ± 0.004 | 0.087 ± 0.009 | 1.415 ± 0.09 |
| 9 | 0.132 ± 0.012 | 0.111 ± 0.020 | 1.205 ± 0.10 |
| 9.5 | 0.108 ± 0.002 | 0.114 ± 0.008 | 0.953 ± 0.06 |
| 10 | 0.037 ± 0.001 | 0.113 ± 0.001 | 0.337 ± 0.01 |

|  | **H172Y** | | |
| --- | --- | --- | --- |
| **pH** | ***k*_cat_ (s^-1^)** | ***K*_m_ (mM)** | ***k*_cat_/*K*_m_ (s^-1^. mM^-1^)** |
| 5.5 | 0.172 ± 0.007 | 0.217 ± 0.019 | 0.537 ± 0.460 |
| 6 | 0.315 ± 0.006 | 0.129 ± 0.005 | 2.44 ± 0.050 |
| 6.5 | 0.418 ± 0.008 | 0.087 ± 0.009 | 4.793 ± 0.440 |
| 7 | 0.592 ± 0.013 | 0.091 ± 0.005 | 6.507 ± 0.025 |
| 7.5 | 0.607 ± 0.005 | 0.083 ± 0.002 | 7.242 ± 0.025 |
| 8 | 0.684 ± 0.032 | 0.094 ± 0.009 | 7.306 ± 0.422 |
| 8.5 | 0.714 ± 0.0189 | 0.100 ± 0.007 | 7.125 ± 0.389 |
| 9 | 0.786 ± 0.062 | 0.123 ± 0.010 | 6.398 ± 0.104 |
| 9.5 | 0.623 ± 0.052 | 0.181 ± 0.014 | 3.439 ± 0.048 |
| 10 | 0.087 ± 0.012 | 0.226 ± 0.043 | 0.387 ± 0.021 |
